# Supplementary material for: Altered Serum Alpha1-Antitrypsin Protease Inhibition before and after Clinical Hematopoietic Stem Cell Transplantation: Association with Risk for Non-Relapse Mortality
Source: Int J Mol Sci. 2023 Dec 28;25(1):422. doi: 10.3390/ijms25010422 (PMC10779144; doi:10.3390/ijms25010422)
Supplement: Supplementary file 1 [file ijms-25-00422-s001.zip › ijms-2759289-supplementary.docx]

**Supplementary Materials and Methods**

**Supplementary Tables**

**Table S1**

Pre-HSCT baseline measurements: serum AAT, CRP, PR3 inhibition, and composite parameter separated by medians (x͂) or clinical cutoff according to eventual acute GVHD grade.

|  | **AAT** | | **CRP** | | **PR3 inhibition** | | **PR3/AAT/CRP ratio** | |
| --- | --- | --- | --- | --- | --- | --- | --- | --- |
| **GVHD grade** | **>**x͂ **N (%)** | **≤**x͂ **N (%)** | **> 3 mg%  N (%)** | **≤ 3 mg%  N (%)** | **>**x͂ **N (%)** | **≤**x͂ **N (%)** | **>**x͂ **N (%)** | **≤**x͂ **N (%)** |
| **1** | 2  (66.6%) | 1  (33.3%) | 0  (0%) | 3  (100%) | 1  (33.3%) | 2  (66.67%) | 1  (33.3%) | 2  (66.7%) |
| **2** | 7  (53.9%) | 6  (46.2%) | 1  (8.3%) | 11  (91. 7%) | 5  (38.5%) | 8  (61.5%) | 5  (41. 7%) | 7  (58.3%) |
| **3** | 3  (60%) | 2  (40%) | 1  (20%) | 4  (80%) | 3  (60%) | 2  (%40) | 2  (40%) | 3  (60%) |
| **4** | 2  (50%) | 2  (50%) | 2  (50%) | 2  (50%) | 3  (75%) | 1  (25%) | 0  (0%) | 4  (100%) |

**Supplementary Figures**

**S1**


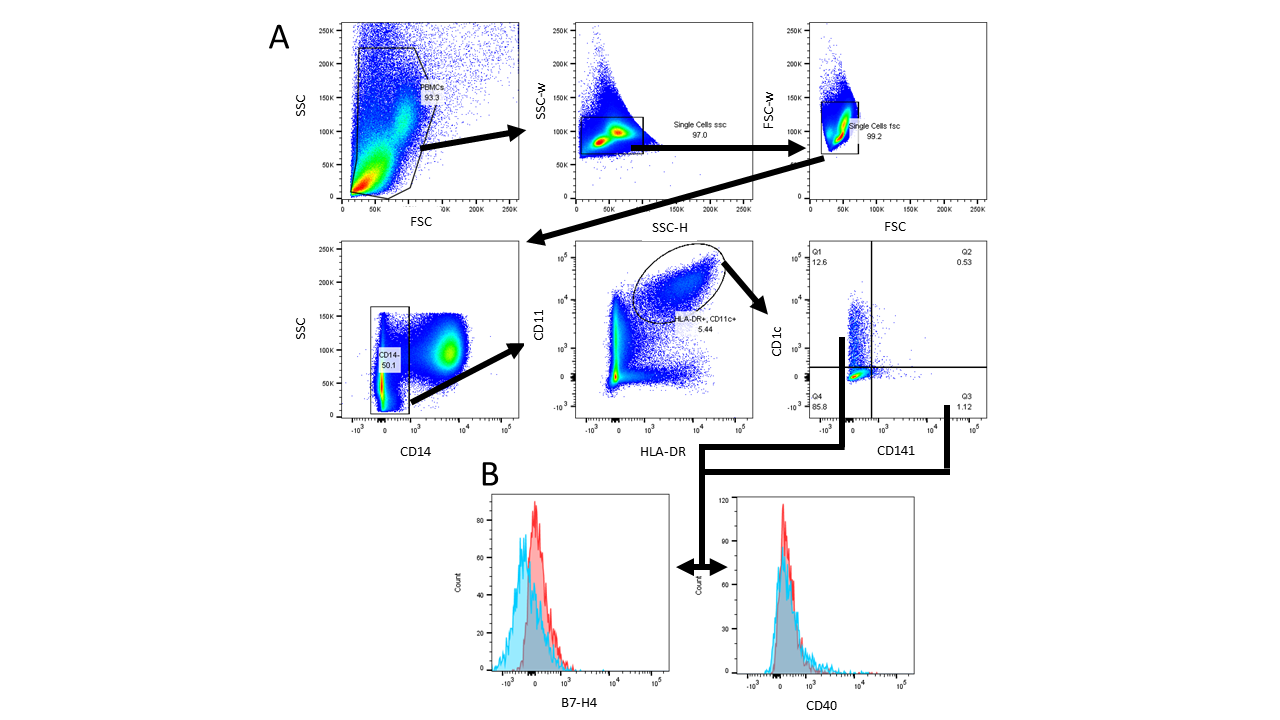


**Figure S1. Myeloid DC subsets gating strategies.** (**A**) Gating strategies for doublets exclusion using side and forward scatters (*top row*) and non-monocytes (CD14^-^) dendritic cells (HLA-DR^+^CD11c^+^) into two myeloid subsets: cDC1 (CD1c^-^CD141^+^) and cDC2 (CD1c^+^CD141^-^) (*bottom row*). (**B**) Expression levels of B7-H4 and CD40 according to fluorescence-minus-one strategy (histograms: *blue*, control; *red*, AAT).

**S2**


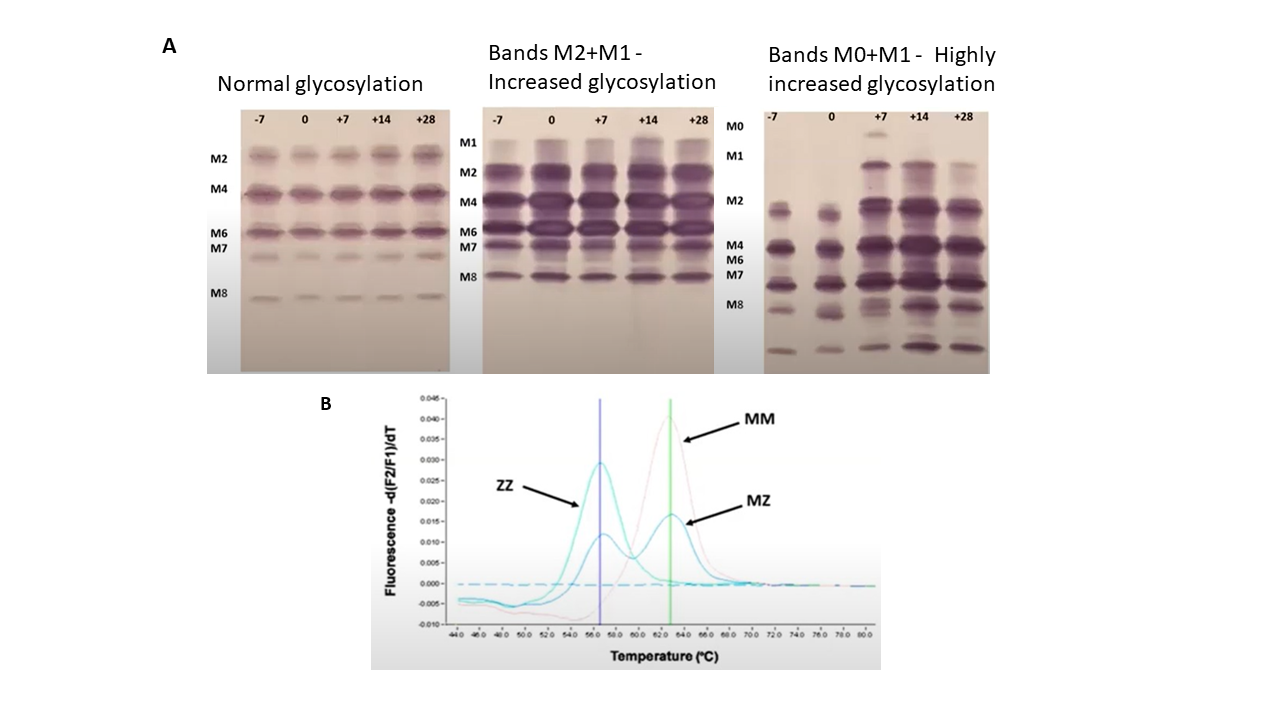


**Figure S2. AAT glycosylation patterns and variant phenotyping*.*** (**A**) *AAT glycosylation patterns*. Representative isoelectric focusing gels from three patients. (**B**) *AAT allele phenotyping*. Representative examples of AAT allele phenotyping of known homozygous and heterozygous phenotypes (MM, common alleles; ZZ, AAT deficiency; MZ, carriers of the Z mutation).

**S3**


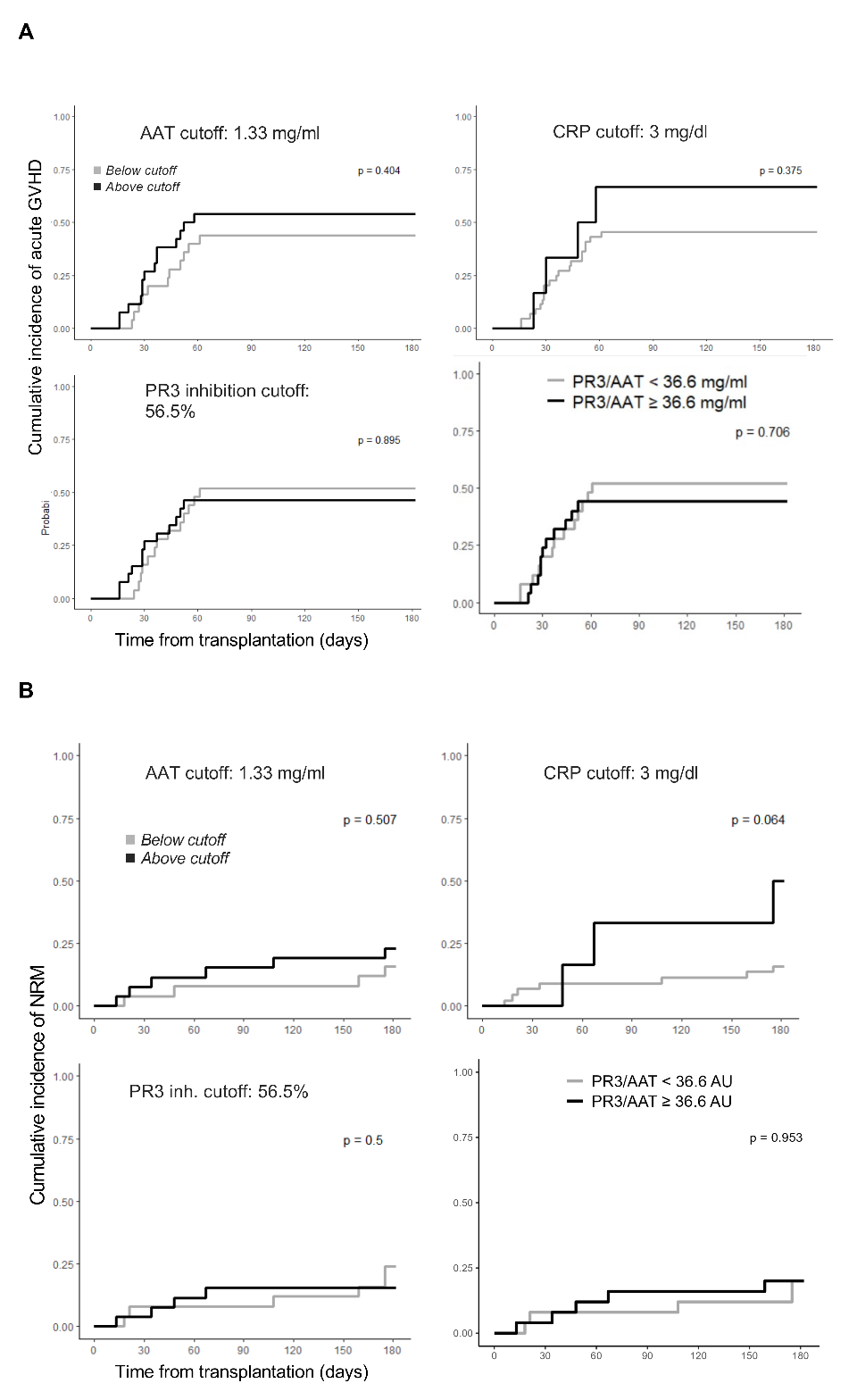


**Figure S3. Cumulative incidence curves of NRM according to AAT and CRP levels, PR3 inhibition and PR3/AAT at baseline measurement**. The probability of developing (**A**) acute GVHD and (**B**) NRM by values of AAT median, CRP clinical cutoff, PR3 inhibition median, and specific PR3 inhibition (PR3/AAT) median. Patient survival compared using Gray’s tests and analyzed with relapse as a competing risk. *p<0.05

**S4**


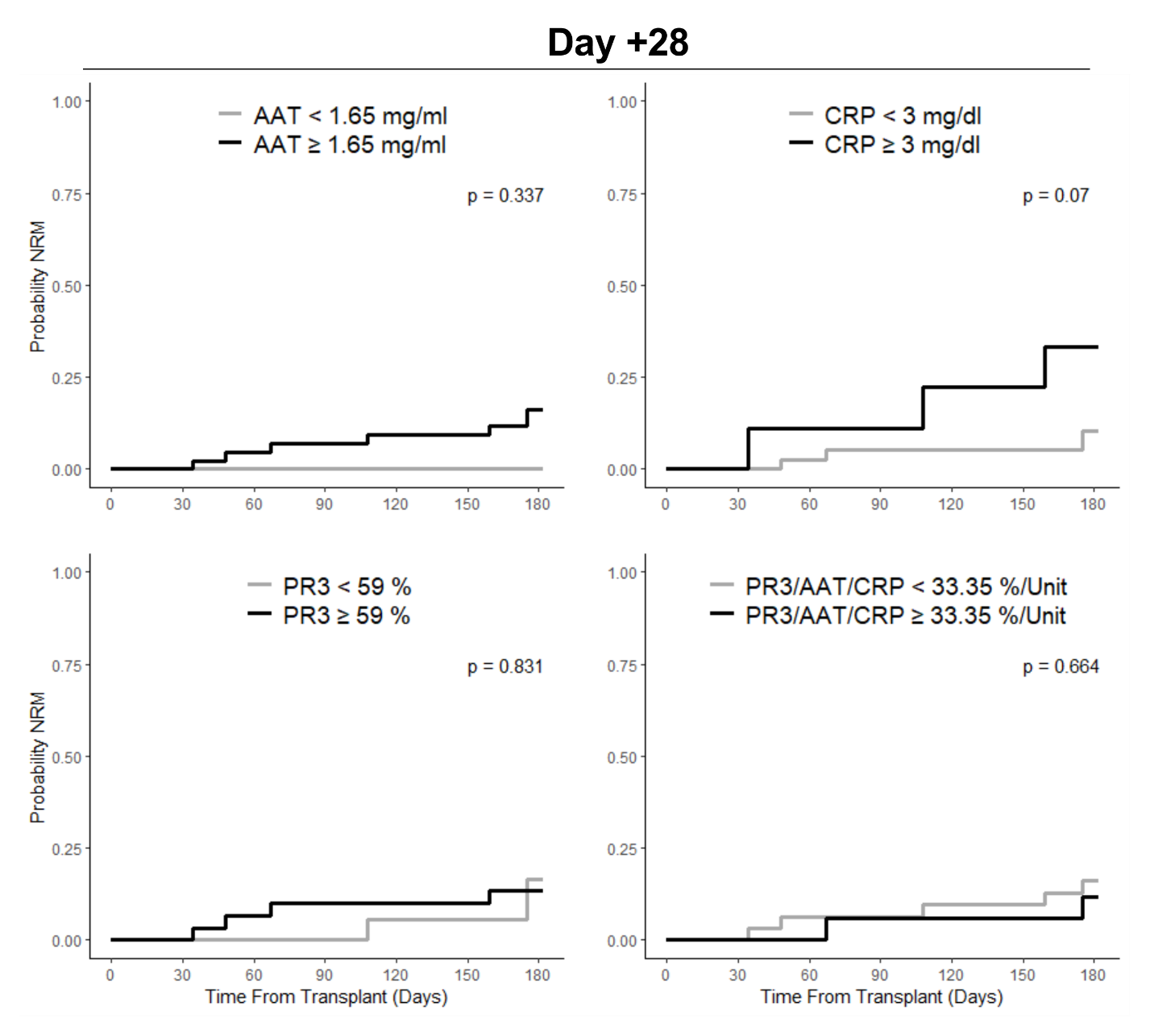


**Figure S4**. **Cumulative incidence curves of NRM according to AAT and CRP levels, PR3 inhibition and PR3/AAT/CRP ratio at day +28 post-transplantation measurement**. NRM according to Day +28 post-HSCT AAT concentration cutoff 1.65 mg/ml (median); CRP concentration cutoff 3 mg/dl (median); PR3 inhibition capacity cutoff 59% (median); and PR3/AAT/CRP ratio cutoff (33.35 AU). Patient survival compared using Gray’s tests and analyzed with relapse as a competing risk. *p<0.05

**S5**

**
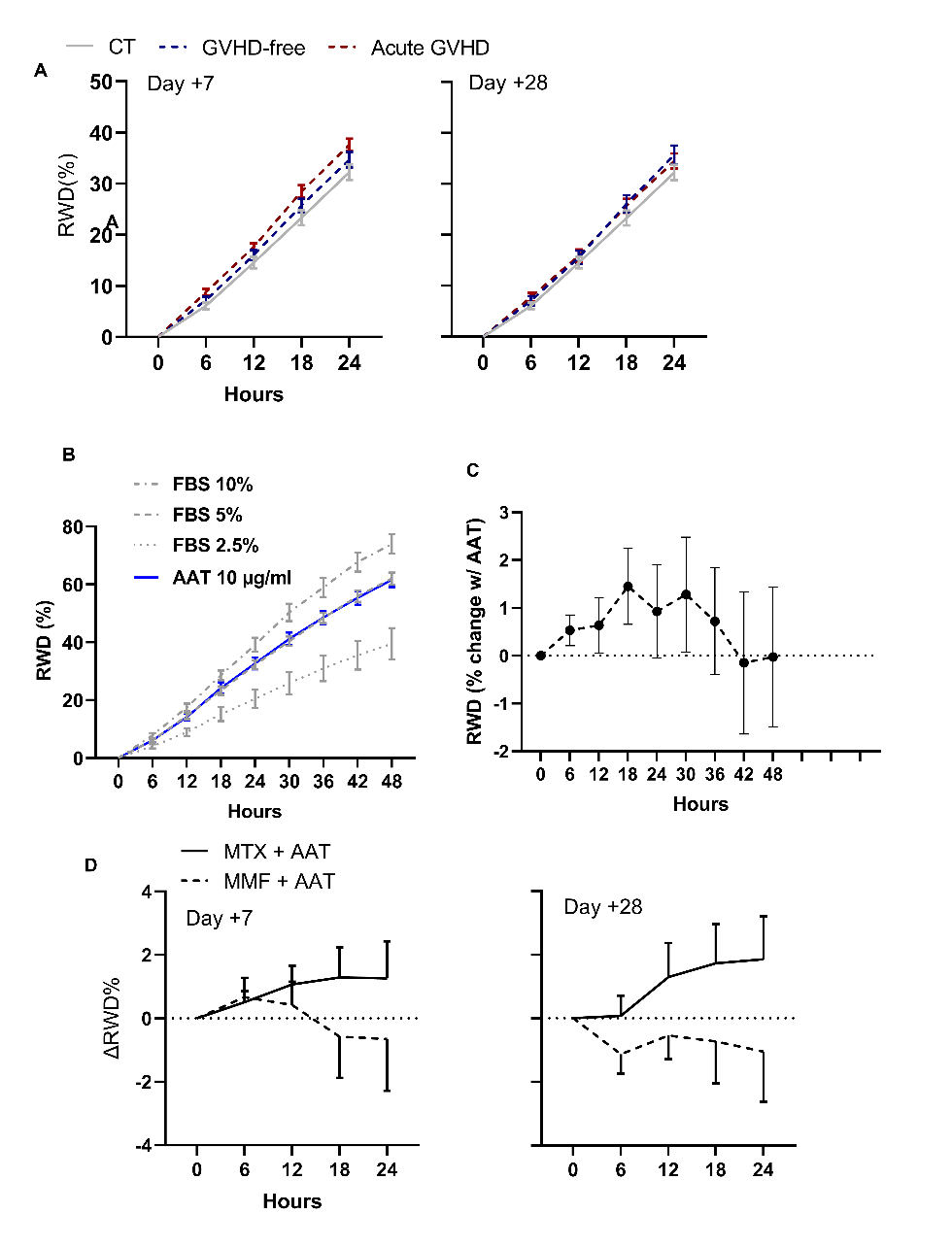
**

**Figure S5. Epithelial cell gap repair controls, added AAT and patient sera response to AAT augmentation: Association with donor and conditioning profiles**. (**A**) Caco2-BBe1 cell monolayers. Patient sera from days 7 and 28 post-HSCT. Re-epithelialization represented by relative wound density (RWD). CT, no added serum, aGVHD and GVHD-free groups. n=22-24 per group.(**B**) Caco2 BBE1 cells supplemented with 10/5/2.5% FBS and AAT (10 μg/ml) or sera collected on days +7 or +28 diluted 1:100 in 5% FBS. (**C**) Change in RWD with addition of AAT (10 μg/ml) from 5% FBS control. Data represent triplicate wells from 8 independent experiments. (**C**) RWD change with added AAT to serum by conditioning protocols (MAC, myeloablative conditioning, n=17; RIC, reduced-intensity conditioning, n=29). (**D**) Change in RWD (ΔRWD) by added AAT (10 µg/ml) distribution by GVHD prophylaxis. MTX, n=30; MMF, n=16. Mean±SEM. Two-way ANOVA with Sidák’s post-hoc test.

**S6**


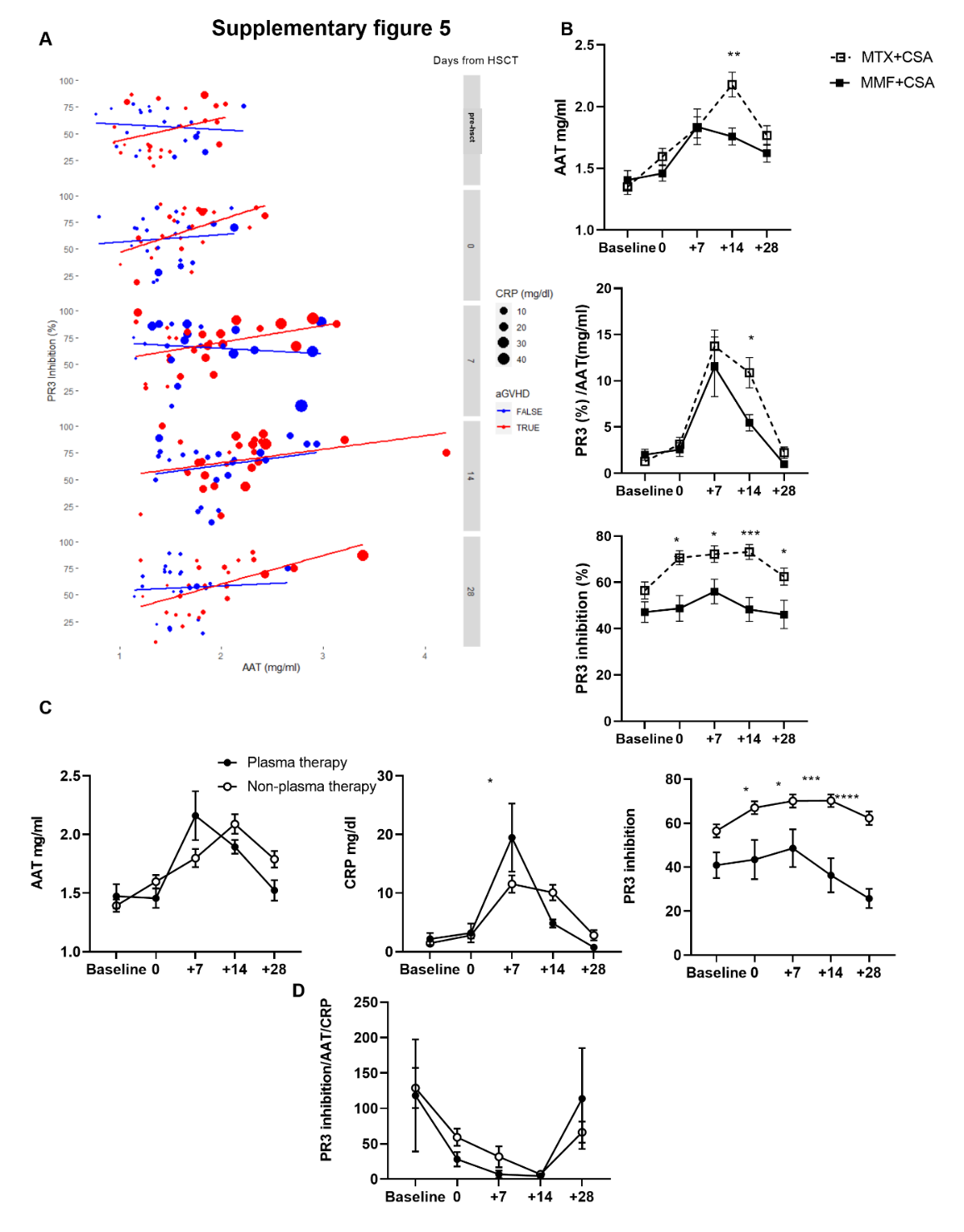

**Figure S6. AAT, CRP and serum PR3 activity, and plasma therapy: aGVHD versus GVHD-free patients**. (**A**) Distribution by individuals of serum levels of AAT, CRP and PR3 inhibition capacity for the acute GVHD group (*red*) and GVHD-free group (*blue*); regression lines for AAT to PR3 activity (n=26 and n=23, respectively). (**B**) Serum levels of AAT, CRP and PR3 inhibition capacity, of patients who received methotrexate-based prophylaxis (MTX, solid circle, n=25-32) and individuals that received mycophenolate mofetil based prophylaxis (open circle, n=17) and (**C**) plasma-treated patients (n=7) and non-plasma-treated patients (n=41-46). (**D**) PR3 specific activity corrected to CRP (calculated as PR3 inhibition/AAT/CRP) in the acute GVHD group vs GVHD-free group (n=26 and n=23 respectively). **(E-F)** Composite values of PR3/AAT/CRP levels and acute GVHD diagnosis according transplantation origin and conditioning regimens; (**E**) PR3/AAT/CRP at each time-point according to matched unrelated donor (MUD) and matched related donor, and acute GVHD diagnosis or GVHD-free status. (**F**) PR3/AAT/CRP at each time-point according to myeloablative conditioning (MAC) and reduced intensity conditioning (RIC), and acute GVHD diagnosis or GVHD-free status. Mean±SEM. Two-way ANOVA with Sidák’s post-hoc test. ns, non-significant, *p<0.05, **p<0.01, ***p<0.001, ****p<0.0001
